# Epidemiology and Mortality of Cryptococcal Disease in Guatemala: Two-Year Results of a Cryptococcal Antigen Screening Program

**DOI:** 10.3390/microorganisms10071388

**Published:** 2022-07-10

**Authors:** Narda Medina, Juan Luis Rodriguez-Tudela, Juan Carlos Pérez, Danicela Mercado, Oscar Bonilla, Eduardo Arathoon, Ana Alastruey-Izquierdo

**Affiliations:** 1 Asociación de Salud Integral, Ciudad de Guatemala 01001, Guatemala; nardagab@gmail.com (N.M.); earathoon@hotmail.com (E.A.); 2 Global Action for Fungal Infections, 1208 Geneva, Switzerland; jlrodrigueztudela@gaffi.org; 3 Clínica Familiar “Luis Ángel García” (Hospital General San Juan de Dios), Ciudad de Guatemala 01001, Guatemala; jcpsmh@gmail.com (J.C.P.); diagnostico@cflag.gt (D.M.); oscar.bonilla@cflag.gt (O.B.); 4 Mycology Reference Laboratory, National Centre for Microbiology, Instituto de Salud Carlos III, 28222 Madrid, Spain

**Keywords:** cryptococcal antigen, meningitis, Guatemala, Latin America, PLWHIV

## Abstract

Cryptococcal disease is an important opportunistic infection among people living with HIV. The cryptococcal antigen (CrAg) can be detected before the clinical onset of meningitis and its screening is recommended. Here, we evaluated CrAg frequency, and describe the epidemiological characteristics and mortality at 180 days in a cohort of HIV patients from Guatemala. A total of 3457 patients were screened with a CrAg lateral flow assay in serum between January 2017 and December 2018. CrAg positivity was 11.5% in patients with ≤100 CD4/mm^3^, 8.7% in patients with <200 CD4/mm^3^, and 6.3% in patients with <350 CD4/mm^3^. In Latin America, we estimated 9.2% CrAg positivity (IC95% 7.9–10.7%) in patients with ≤100 CD4/mm^3^. Among patients newly diagnosed with HIV, we estimated 4416 incident cases per year in Latin America in those with <200 CD4/mm^3^ and 5289 in those with <350 CD4/mm^3^. In addition, we calculated the burden in people not on ARV or without viral suppression and found 28,672 cases. CrAg screening should be considered in patients who have a CD4 cell count < 350 cells/mm^3^. Cryptococcal meningitis was associated with 30.8% mortality in Guatemala. Global access to diagnosis as well as to liposomal amphotericin B and flucytosine is a priority.

## 1. Introduction

Cryptococcal disease remains a major cause of opportunistic infections that affect people living with HIV (PLWHIV). The World Health Organization (WHO) guidelines recommend screening for cryptococcal antigen (CrAg) followed by pre-emptive antifungal therapy among CrAg-positive people with a negative cerebrospinal fluid (CSF) test to prevent the development of cryptococcal meningitis and before initiating or reinitiating antiretroviral therapy (ART) [1]. These guidelines advise CrAg screening in patients with <200 CD4/mm^3^ [1]. In 2017, an update of global cryptococcosis estimated that there were 278,000 people positive for cryptococcal antigen in people with <100 CD4/mm^3^ [2]. Cryptococcal meningitis causes ≈15% of HIV-related deaths [2]. Thus, rapid and accurate laboratory diagnosis is critical to initiate antifungal treatment in a timely manner, which is correlated with improved survival. 

The cryptococcus lateral flow assay (CrAg LFA) has a sensitivity and specificity of >98% [3,4,5]. Minimal laboratory infrastructure is required to perform this test and results are available after 10 min. Several countries have implemented projects to screen for cryptococcal disease among PLWHIV. The overall prevalence of CrAg in HIV patients with <100 CD4/mm^3^ has been reported at 6.0% (95% CI 5.8–6.2) [2]. However, much of the data are based on studies performed outside of the Latin America region. Epidemiological data of cryptococcal disease is essential for health systems. They help to organize where the diagnostic resources should be located, in primary care or in more specialized settings, as well as the size and cost of the intervention required. In this study, we analyzed the results of a two-year CrAg screening program in Guatemala and compare them with those of previously published studies. Further, we analyzed the epidemiological characteristics of patients and 6-month mortality rate. 

## 2. Materials and Methods

### 2.1. Setting and Study Design

The opportunistic infection program implemented in Guatemala officially started in January 2017. This program encompasses data from 13 health care facilities that provide services for PLWHIV by means of a Diagnostic Laboratory Hub. Specific details of the program have been published [6,7]. Briefly, three groups of patients were enrolled: (i) patients newly diagnosed with HIV; (ii) patients who have not been receiving antiretroviral treatment (ART) for >90 days but who returned to care (Return/Restart); and (iii) patients on ART with symptoms of opportunistic infections (on ARV treatment). Patients were screened for tuberculosis (TB), non-tuberculous mycobacteria (NTM), histoplasmosis, and cryptococcal disease, regardless of their CD4 cell count and symptomatology. CrAg LFA screening was performed on serum samples at the health care facilities whilst other determinations were performed at the Diagnostic Laboratory Hub. Demographic data were collected using a standard electronic form. An audit and a quality control program were set up to ascertain the results provided by the health care facilities. One in every five serum samples was tested with a CrAg LFA at the Diagnostic Laboratory Hub for results confirmation.

### 2.2. Literature Review

A literature search was performed in May 2022 using PubMed, Scielo, and GoogleScholar to identify published studies in Latin America in which the CrAg frequency in serum was reported. Keywords included “cryptococcal antigen”, “cryptococcal infection”, “cryptococcal disease”, “HIV patients”, “people living with HIV”, “Latin America”, “Central America”, and “South America”. Other studies were identified using snowballing. According to these studies, and our results, we estimated the frequency of cryptococcal antigenemia in the region. 

To calculate the burden of cryptococcal disease in Latin America, we used data published by UNAIDS [8]. To estimate the number of patients at high risk of cryptococcal disease in Latin America, we proposed the following scenario: (i) all patients were screened regardless of symptoms and CD4 counts, and we compared the % CrAg positivity in Guatemala to that of every country with data provided by UNAIDS for patients newly diagnosed with Advanced HIV Disease (AHD), defined as <200 CD4/mm^3^. (ii) We calculated that 55% of patients have <350 CD4/mm^3^ in the region and we applied the % CrAg positivity that we found in the Guatemalan cohort [7]. (iii) We also used UNAIDS data to calculate the number of PLWHIV who are not on ART and those who are not virally suppressed per country in 2020 [8]. Then, we found that 20% of patients in these two groups would have AHD, which is a more conservative figure than the average found in patients newly diagnosed with HIV (≈30%) (see Supplementary Material). 

### 2.3. Statistical Analysis 

The statistical analysis was performed using SPSS (IBM Iberica, Madrid, Spain). Baseline characteristics were compared with the chi-square test or Fisher’s exact test for categorical variables. Frequencies were calculated from patients tested globally and for each group of patients using percentages. Mortality among cryptococcal cases was analyzed with the Kaplan–Meier test at 180-day follow-up. A *p* value of <0.05 was considered statistically significant.

## 3. Results

We screened a total of 3457 patients with a CrAg LFA in serum between January 2017 and December 2018. These patients were enrolled as part of an opportunistic infection program implemented in Guatemala as described above. Among cryptococcal cases, the median age at screening was 38 years [IQR: 32–44 years], 71.3% (n = 117) were males, and 48.8% (n = 80) were from urban areas. Cryptococcosis was associated with men (*p =* 0.0364). Patients with cryptococcosis were slightly younger than patients without the disease (median 34 vs. 38 years old, *p <* 0.0001). Cryptococcal cases had significantly lower CD4 counts than non-cryptococcal cases (median CD4, 47 cells/mm^3^ vs. 213 cells/mm^3^; *p* < 0.0001). Multiple opportunistic infections were diagnosed in thirty-two patients (19.5%). The most frequent combinations were cryptococcosis plus histoplasmosis 15 (46.8%) and cryptococcosis plus tuberculosis 11 (34.2%). Table 1 summarizes patients’ characteristics.

At enrollment, CD4 cell counts were available for 2589 patients (74.9%). The incidence of CrAg in patients with ≤100, <200 and <350 cells/mm^3^ was 11.5%, 8.7%, and 6.3%, respectively (Table 2). Patients newly diagnosed with HIV had the highest CrAg incidence in patients with ≤100 cells/mm^3^ compared to patients on ARV and those who return to care (Table 2) but this result was not statistically significant (*p =* 0.5427). 

**Figure 1:**
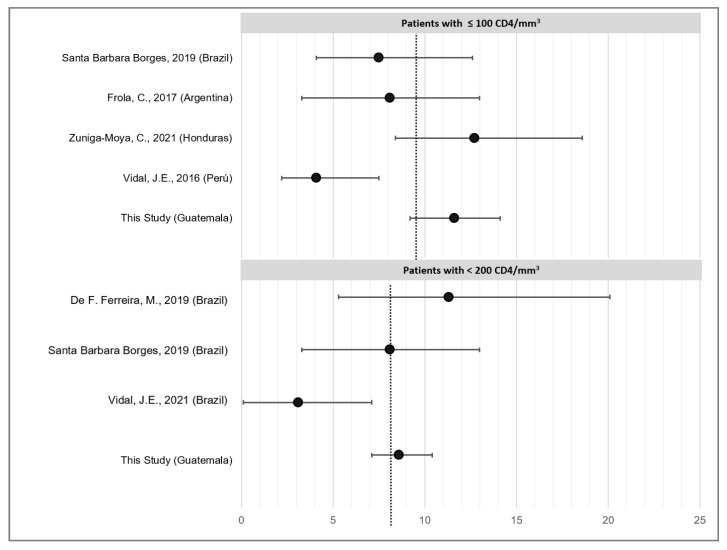
Frequencies of cryptococcal antigenemia in LATAM among patients with ≤100 cells/mm^3^ and those with AHD (<200 CD4/mm^3^) [9,10,11,12,13,14].

In other Latin American countries, published studies showed that CrAg positivity ranged from 8.1% to 12.7% in patients with <100 cells/mm^3^ and from 7.9% to 11.2% in patients with AHD (references) (Figure 1). For more information about the reporting criteria in each country, see Supplementary Data (Supplementary Material). Considering all regional publications, four reported the frequency of CrAg in patients with ≤100 cells/mm^3^ [9,10,11,12] and three in patients with <200 cells/mm^3^ [9,13,14]. Using these results and ours, the estimated overall CrAg frequency in the region for PLWHIV with <100 CD4 cells/mm^3^ was 9.2% (IC95% 7.9–10.7%) and 8.1% in those with AHD (IC95% 7.9–10.7%). 

According to UNAIDS, in 2020, 2.1 million of people were living with HIV in Latin America and 109,030 cases were newly diagnosed with HIV [8]. Considering the burden of AHD per country (Supplementary Material), 2828 incident cases per year are expected; however, if we take into account that 55% had <350 CD4 cells/mm^3^, the yearly incidence in the region would rise to 3778 cases. This means that if we only screen AHD patients, 950 cases will be missed every year. For other at-risk groups, such as those who abandon ART or those on ART, we considered UNAIDS data on the number of patients without ART treatment as well as those who are not virally suppressed. Then, we considered that 20% of them would have AHD (Supplementary Material), which means that 13,205 and 15,466 cases of cryptococcosis could be expected in those at-risk groups, respectively. 

In Guatemala, only 91 patients (55.4%) with a positive serum CrAg result had a lumbar puncture to rule out meningeal involvement. Sixty (65.9%) had cryptococcal meningitis. In a short survey, the health care facilities participating in the program stated that the main reasons for not performing a lumbar puncture were: patients’ refusal, lack of medical supplies, and clinical contraindications to performing a lumbar puncture. 

**Figure 2:**
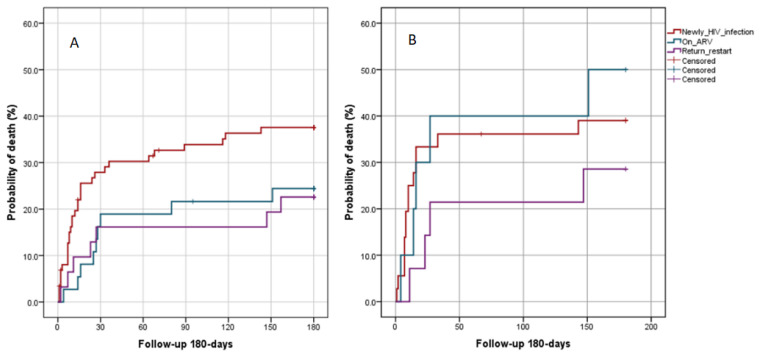
(**A**) Overall mortality among patients with a positive CrAg serum. (**B**) Overall mortality among patients with a positive CrAg serum and cryptococcal meningitis.

One hundred and fifty-six cryptococcal cases were followed up at 180 days in this study; of these, 48 deaths (30.8%) were reported. Thirty-six (75%) of these deaths occurred in the first 30 days. Figure 2 shows the overall probability of death in patients with cryptococcal antigenemia and those with cryptococcal meningitis per group of patients. Among patients with positive serum antigenemia, the highest mortality was found in patients newly diagnosed with HIV, with 36.4% (32 out of 88). In patients who return to care and those on ART, mortality was 22.6% and 24.3%, respectively. AHD patients had a higher mortality than non-AHD patients (31.8% vs. 20%, *p =* 0.322). Regarding cryptococcal meningitis, overall mortality at 180 days of follow-up was 38.3% (23 out of 60). Fifty percent (5 out of 10) of patients on ART died, whilst mortality was 28.6% and 38.9% in patients who return to care and among patients newly diagnosed with HIV, respectively. In patients who had a positive serum antigen but did not have a lumbar puncture, overall mortality was 30.3%. On the other hand, mortality in those with a negative CrAg lumbar puncture was 16.7% (5 out of 30, *p =* 0.150). Among these cases, three had coinfections, one had tuberculosis and two had histoplasmosis. It is important to take into account that although liposomal amphotericin B and flucytosine are recommended as antifungal treatments, none were available in Guatemala during the study period. Amphotericin B deoxycholate and fluconazole were the drugs used for treatment.

## 4. Discussion

Among Latin American people with HIV, we estimated that CrAg positivity is 9.2% (IC95% 7.9–10.7%) in patients with <100 CD4/mm^3^ [9,10,11,12,13,14]. This number is 3.2% higher than the global estimation by Rajasingham et al. in asymptomatic patients [2], which is an uncertain denomination considering the deep immunosuppressed status of such HIV patients. In published articles from Brazil, Argentina and Honduras, the criteria to establish the burden of cryptococcal disease were similar, with CD4 thresholds of <100 or <200 cells/mm^3^ [10,11,13]. However, in Guatemala, we screened patients regardless of the CD4 cell count, symptoms, or hospitalized status, with an overall incidence of 4.8% (Table 2), which is a real measurement of the burden of disease in PLWHIV. The burden of disease is key to planning how and where health resources should be allocated depending on the frequency of the disease and the complexity of diagnosis. Therefore, it is important to analyze the study criteria to avoid underestimations of the burden of disease. In at high-risk populations, such as patients with AHD, screening based on symptoms can be biased due to the noise [15] that different clinicians with different training and judgment can introduce in patient selection. Evaluating the absence or the presence of symptoms in highly immunosuppressed populations is a risky exercise. For instance, only asymptomatic hospitalized patients were included in the study by Vidal et al. [13], where only a 3.1% incidence was ascertained, and this may mean that patients had another diseases that caused hospitalization and cryptococcal cases diagnosed were comorbidities more than a burden of this disease in asymptomatic people at risk. Thus, to establish the burden of disease, we can choose an objective entry criterion, such as CD4 threshold. However, to calculate the real burden of disease, studies analyzing the whole population at risk of the disease must be performed. In this case, for HIV, we stratified by risk factors (e.g., CD4 threshold). In Table 2, we show the number of CrAg-positive patients according to CD4 counts, based on which we can decide whether to introduce screening strategies and at what threshold. 

One of the most striking results of this analysis is the burden of cryptococcosis disease in patients who return to ARV therapy and those on ARV but with unsuppressed viral loads. The estimation of the burden of cryptococcal disease in PLWHIV and those with AHD could reach 28,000 cases. Considering the mortality rate observed in Guatemala at 180 days, we estimate that ≈8600 patients will die in Latin America due to a life-threatening disease which is easy to diagnose with a CrAg LFA. In Botswana, despite the wide availability of HIV testing and treatment services, a study showed that there was a substantial burden of cryptococcal meningitis due to a failure to effectively engage or retain patients in care [16]. Several studies have described the barriers that face patients who return to care after loss to follow up [17,18]. As cryptococcal disease will not go away without treatment and can lead to meningitis and eventually death, it is possible that many such patients die before diagnosis. 

The CrAg LFA has a sensitivity and specificity of >98% [5]. In Guatemala, a comparative performance analysis found that 97% of cryptococcal cases were diagnosed by this assay [19]. This test has been included in the WHO essential diagnostics list since 2017 [20]. However, in many low- and middle-income countries, this test is not yet available. Concerning when to screen for CrAg, our study has an advantage in that the test was performed regardless of the CD4 count. Screening patients for AHD disease is unrealistic unless the clinician has the CD4 count as soon as the patient arrives. It is clear and straightforward that quickly knowing the CD4 count reduces the bias and noise of different clinical criteria. However, in many places, it is difficult to obtain this result. In Guatemala, only 74.9% of patients had their CD4 count available at evaluation. In addition, most of the studies and guidelines have focused on the benefit of CrAg screening at CD4 cell counts <100 or on AHD. However, the results of the Guatemala screening shows that 95% of the cryptococcal cases were diagnosed at <350 CD4/mm^3^, which is 8% higher than those diagnosed at <200 CD4/mm^3^. If we extrapolate our results for patients newly diagnosed with HIV in Latin America, we estimate that using a threshold of 200 CD4/mm^3^, 950 cases would have been missed in comparison to using a threshold of <350 CD4/mm^3^. Overlooked detection of positive cases has severe consequences because CrAg positivity is an independent predictor of meningitis and death [5]. In Guatemala, in 2021, each CrAg test cost USD 7 and USD 2 per pre-emptive treatment with fluconazole. However, in Africa, the total cost associated with one case of cryptococcal meningitis is estimated at USD 2125 [21]. According to our results (Table 2), we recommend performing CrAg screening for all patients with <350 cells/mm^3^. Of course the clinician’s judgment must prevail when the CD4 count is not available at evaluation but taking into account that cryptococcosis is a life-threatening disease, a CrAg LFA is cheap, easy to perform and provides a result within ten minutes, which allows making health interventions based on evidence.

Concerning lumbar puncture, the WHO guidelines recommend performing a lumbar puncture to rule out meningitis in any CrAg LFA-positive patient [1]. However, in some settings, access to lumbar punctures may be difficult, in addition to the fact that some patients will refuse this procedure. Here, only 55.4% of patients with a CrAg-positive result received a lumbar puncture, of whom 65% had meningitis. Thus, it is certain that some meningeal cases were not diagnosed. The use of a CrAg LFA needs to be reinforced because of the high mortality rate of cryptococcal meningitis. In the future, the use of a semiquantitative test and artificial intelligence algorithms for CrAg interpretation may help to identify those at high risk of meningitis and death [22,23,24]. Early cryptococcal diagnosis is key to decreasing mortality rates. In this study, the 180-day probability of death in patients with a CrAg-positive result was 30.8%. Three-quarters of those deaths occurred within 30 days of diagnosis. As expected, mortality in patients with cryptococcal meningitis was higher than in non-meningeal cases (38% vs. 16.7%); however, although mortality in non-meningeal cases was substantial, the co-occurrence of other infections may contribute to it. Three out of five (60%) patients had coinfections—one tuberculosis and two histoplasmosis—which highlights the importance of screening programs that provide a rapid diagnosis for the most frequent opportunistic infections. 

The current recommended treatment for cryptococcal meningitis includes amphotericin B deoxycholate and flucytosine, followed by fluconazole [1]. It is important to highlight that liposomal amphotericin B is preferred since it has demonstrated equivalent efficacy and better safety and tolerability than deoxycholate formulation. The principal barriers for this liposomal amphotericin B plus flucytosine regimen are its availability and cost. Data from the Global Action for Fungal Infections (GAFFI) showed that only three countries in the region had flucytosine [25]. Additionally, the daily price of fluconazole varied from <USD 1 to USD 31 [26]. Despite new approaches such as the use of a single high dose of liposomal amphotericin B plus flucytosine and fluconazole showing a non-inferior response and fewer adverse events [27], access to these treatments is mandatory.

This study has limitations. The relatively low number of studies in the region and the lack of estimates concerning different groups of people living with HIV who are at high risk of cryptococcal disease could introduce changes in our estimates. Lack of detailed information regarding symptoms and ART adherence, which might influence outcomes, is not available. Furthermore, we define deaths in the cryptococcal disease group as attributable to this pathogen; however, patients with HIV could have additional comorbidities. Despite these limitations, this study shows a substantial burden of cryptococcal disease among people living with HIV in Latin America in addition to a high mortality rate, especially in those with cryptococcal meningitis and those without a lumbar puncture. Estimations by Rajasingham et al. (2) suggest a burden of 7000 (3600–11,100) cases for Latin America in PLWHIV with <100 CD4/mm^3^. Our analysis shows that the burden of cryptococcal disease among those with AHD in the region is substantially higher, reaching a prevalence of 31,503 cases. Although we calculated the numbers of cases for PLWHIV with AHD, the incidence of cryptococcal disease in Latin America for those with <100 CD4/mm^3^ is 3.2% higher than the global estimation, meaning that an underestimation of the burden of this life-threatening disease in this region is highly probable. There is very robust data for the Guatemalan cohort because of the number of patients included and the prevalence of cryptococcal disease in patients with <100 CD4/mm^3^ is 11.5%, almost 2-fold that reported for the global population (2), which further supports the estimations for this region. Optimal treatment of cryptococcal meningitis requires the integration of lumbar puncture as well as the availability of liposomal amphotericin B and flucytosine to reduce AIDS-related deaths.

## Figures and Tables

**Table 1:** Baseline characteristics of patients screened using a CrAg LFA.

Characteristics	CrAg + ve		CrAg − ve		Patients Screened	
	N	%	n	%	n	%
Gender						
Male	117	71.3%	2082	63.2%	2199	63.6%
Female	46	28.0%	1175	35.7%	1221	35.3%
Transsexual	1	0.6%	36	1.1%	37	1.1%
Sexual orientation						
Heterosexual	129	78.7%	2427	73.7%	2556	73.9%
Homosexual	16	9.8%	599	18.2%	615	17.8%
Bisexual	7	4.3%	205	6.2%	212	6.1%
Ethnic group						
Ladino	126	76.8%	2404	73.0%	2530	73.2%
Mayan	12	7.3%	480	14.6%	492	14.2%
Other	2	1.2%	19	0.6%	21	0.6%
Unknown	24	14.6%	390	11.8%	414	12.0%
Rural						
Yes	74	45.1%	1565	47.5%	1639	47.4%
CD4 cell count						
Unknown	39	23.8%	829	25.2%	868	25.1%
<50	66	40.2%	397	12.1%	463	13.4%
50–99	21	12.8%	265	8.0%	286	8.3%
100–199	22	13.4%	486	14.8%	508	14.7%
200–349	10	6.1%	612	18.6%	622	18.0%
≥350	6	3.7%	704	21.4%	710	20.5%

**Table 2:** Incidence of CrAg in serum according to the CD4 count in Guatemala.

Characteristics	CD4 Threshold (Cells/mm^3^)				Overall Incidence
	≤100	<200	<350	≥350	
Newly diagnosed with HIV	12.3%	8.8	6.4	0.3	5.0
On ARV	10.1%	8.8	6.8	1.3	4.6
Return to the ARV	10.5%	8.2	5.7	1.6	4.6
Total	11.5%	8.7	6.3	0.8	4.8

## Supplementary Materials

The following supporting information can be downloaded at: https://www.mdpi.com/article/10.3390/microorganisms10071388/s1, Table S1. Cryptococcal antigenemia prevalence studies in LATAM; Table S2. Estimates of CrAg in people living with HIV in Latin America.
