# Supplementary material for: Epidemiology and Mortality of Cryptococcal Disease in Guatemala: Two-Year Results of a Cryptococcal Antigen Screening Program
Source: Microorganisms. 2022 Jul 10;10(7):1388. doi: 10.3390/microorganisms10071388 (PMC9323764; doi:10.3390/microorganisms10071388)
Supplement: Supplementary file 1 [file microorganisms-10-01388-s001.zip › microorganisms-1796139-supplementary.pdf]

Supplementary Material

Table S1. Cryptococcal antigenemia prevalence studies in LATAM

Author	Years of study	Enrolled patients	Country	Criteria for serum CrAg report	Serum CrAg positivity
**Patients with ≤ 100 CD4/mm^3**					
Santa Barbara Borges, M., *et al.* [9]	2015-2018	214	Brazil	Patients with ≤100 cells/mm^3 without previous cryptococcal disease and regardless of symptoms	7.5%
Frola, C., *et al.* [10]	April 2014 to January 2015	123	Argentina	HIV patients with CD4 ≤100cells/mm^3 within 3 months or WHO stage III/IV	8.1%
C. Zuniga-Moya [12]	2017-2018	220	Honduras	HIV patients who had CD4 ≤100 cells/mm^3	12.7%
Vidal, J.E., *et al.* [12][1]	----	368	Perú	ART-naïve adults with CD4 of ≤100 cell/mm^3 without a history of cryptococcosis	3.6%
**Patients with < 200 CD4/mm^3**					
Santa Barbara Borges, M., *et al.* [9]	2015-2018	214	Brazil	AHD without previous cryptococcal disease and regardless of symptoms	7.9%
De F. Ferreira, M., *et al.* [13]	2015	89	Brazil	hospitalized HIV patients with AHD	11.2%
Vidal, J.E., *et al.* [14]	November 2014 to 30 October 2015	163	Brazil	hospitalized HIV patients with AHD without symptomatic meningitis	3.1%

[1] This study reported data of another program *serum sample

Table S2. Estimates of CrAg in people living with HIV in Latin America

Formulas used in this analysis:

A. *Newly HIV diagnosed at risk of cryptococcal disease with AHD:* [(New HIV diagnoses*) (% AHD reported*)] (CrAg=8.7%)
B. *Newly HIV diagnosed at risk of cryptococcal disease with <350 CD4/mm^3:* [(New HIV diagnoses*) (55%)] (CrAg=6.3%)
C. *Abandon the ART:* [(Estimated No. Of people NOT on ART) (20%)] (CrAg=8.7%)
D. *On ART:* [(Estimated No. NOT virally suppressed) (20%)] (CrAg=8.7%)

Countries	PLWH 2020*	New HIV diagnoses*	% AHD reported*	% PLWH who are on ART*	Estimated No. Of people NOT on ART	%PLWH who are virally suppressed*	Estimated No. NOT virally suppressed
Argentina	140,000	5,600	24	65	49000	55¥	63000
Bolivia	17,000	870	30¥	68	5440	55¥	7650
Brazil	930,000	48,000	27	70	279000	66	316200
Chile	77,000	5,000	30	70	23100	63	28490
Colombia	180,000	9,300	35	64	64800	57	77400
Costa Rica	16,000	1,700	30¥	53	7520	55¥	7200
Ecuador	45,000	2,100	18	76	10800	55¥	20250
El Salvador	25,000	850	25	56	11000	49	12750
Guatemala	33,000	770	52*	64	11880	56	14520
Honduras	22,000	710	30¥	54	10120	31	15180
Mexico	340,000	20,000	39	55	153000	49	173400
Nicaragua	12,000	740	22	50	6000	55¥	5400
Panama	31,000	1,800	38	51	15190	53	14570
Paraguay	19,000	890	44	53	8930	44	10640
Peru	91,000	4,100	16	79	19110	55¥	40950
Dominican Republic	75,000	3,400	30¥	51	36750	55¥	33750
Uruguay	12,000	1,000	10	81	2280	79	2520
Venezuela	100,000	2,200	30¥	55	45000	55¥	45000

**UNAIDS 2020 Data[1];*Guatemala data[2];¥ data based on the overall average (% of AHD= 30%; %virally suppressed=55%)*

[1] Unaids. (n.d.). UNAIDS data 2021.

[2] Medina, N., Alastruey-Izquierdo, A., Bonilla, O., Gamboa, O., Mercado, D., Pérez, J. C., Salazar, L. R., Arathoon, E., Denning, D. W., & Luis Rodriguez-Tudela, J. (2021). A Rapid Screening Program for Histoplasmosis, Tuberculosis, and Cryptococcosis Reduces Mortality in HIV Patients from Guatemala.
